# B49, a BST-2-based peptide, inhibits adhesion and growth of breast cancer cells

**DOI:** 10.1038/s41598-018-22364-z

**Published:** 2018-03-09

**Authors:** Wadie D. Mahauad-Fernandez, Chioma M. Okeoma

**Affiliations:** 1 Department of Microbiology and Immunology, Carver College of Medicine, University of Iowa, 51 Newton Road, Iowa City, IA 52242–1109 USA; 2 Present Address: Division of Oncology, Departments of Medicine and Pathology, Stanford University School of Medicine, 291 Campus Drive, Stanford, CA 94305 USA; 3 Present Address: Department of Pharmacology, Stony Brook University, 101 Nicolls Rd, Stony Brook, NY 11794–8651 USA

## Abstract

Bone marrow stromal antigen 2 (BST-2) also known as Tetherin has been implicated in the growth and progression of many cancers. BST-2 employs its pro-tumor effects through the formation of BST-2:BST-2 dimers which ultimately promotes cell to cell and cell to matrix adhesion, cell motility, survival, and growth. The aim of this study was to evaluate the effect of a novel BST-2-based peptide—B49 on adhesion and growth of breast cancer cells. Homotypic/heterotypic adhesion, three-dimensional spheroid formation, and anchorage-independent growth were used to assess the effect of B49 on cell adhesion and growth. Additionally, we provide evidence of the anti-tumor effect of B49 in a preclinical mouse model of breast cancer. Results show that breast cancer cell adhesion to other cancer cells or components of the tumor microenvironment were inhibited by B49. Most well-known evaluation indexes of cancer cell growth, including spheroid formation, anchorage-independent, and primary tumor growth were significantly inhibited by B49. These data affirm that i) BST-2 plays a key role in mediating breast cancer cell adhesion and growth, and ii) B49 and its analog B49Mod1 significantly inhibits BST-2-mediated cancer cell adhesion and growth. Therefore, B49 and its analogs offer a promising anti-adhesion and therapeutic lead for BST-2-dependent cancers.

## Introduction

Breast cancer is the second largest cause of cancer-related deaths in women, accounting for over 450,000 deaths per year worldwide. Over the last 15 years, the treatment of breast cancer has evolved to include therapies aimed at specific molecular subtypes of the disease^1^. Five distinct subtypes (Luminal A, Luminal B, HER2 enriched, basal, and claudin low) have become increasingly recognized to have clinical significance^1–3^. In these classes, some tumor types have become easier to treat with the advent of specific biological markers and drugs aimed at alterations within a subtype. A notable advance is the recognition of the receptor protein-tyrosine kinase erb-B2 (HER2) positive subtypes, which can be targeted by anti-HER2 antibodies such as trastuzumab (Herceptin, Genentech)^4^. Interestingly, the protein BST-2 (also called tetherin, CD317 and HM1.24) is elevated in various tumors and cancer cells with no subtype specificity, at least in breast cancer^5^. In breast tumors, the level of BST-2 is significantly higher when compared to notable markers of breast cancer, including estrogen receptor, progesterone receptor, HER2, or Myc^6^.

It was within this context that we became interested in how the expression of BST-2 might be playing a role in breast cancer. The roles of BST-2 in inhibiting viral release^7–9^, promoting cell to cell virus transmission through the formation of viral clusters^10^, and in promoting breast cancer^6,11^, appear to be linked to its structure, especially the covalent bonds between cysteine residues in the extracellular domain of BST-2^6,12–14^. BST-2 is a membrane-tethered glycoprotein expressed on the cell surface^15^ and aberrantly expressed in various mouse and human tumors^5,16–21^. The N-terminus of the human BST-2 extracellular domain comprises three cysteine residues located at positions 53, 63, and 91 that orchestrate formation of covalent cysteine-linked BST-2 homodimers^22^.

The significance of BST-2 cysteine-linked dimerization in breast cancer was not appreciated until we showed that the extracellular domain cysteine residues, charged with orchestrating BST-2 dimerization promotes BST-2-directed cell to cell and cell to extracellular matrix (ECM) interaction, anoikis resistance, cell survival, and tumor growth^6^. We further showed that the mechanism by which BST-2 dimerization promotes breast cancer involves a previously unreported BST-2/GRB2/ERK/BIM/Cas3 pathway^6^. These data point to the BST-2 extracellular domain as a druggable target and provide proof of principle for a potential therapeutic approach based on interfering with BST-2-mediated cell to cell or cell to ECM interactions.

Our previous study provides evidence that disruption of BST-2 dimerization prevents adhesion of breast cancer cells to each other, to immune cells, and to ECM substrates^6^. The loss of BST-2 dimerization-mediated cell to cell/ECM interaction inhibits cancer cell clustering, induces anoikis in breast cancer cells through BST-2/GRB2/ERK/BIM/Cas3 pathway, and inhibits tumor growth and metastasis^6^. On the basis of these findings, we hypothesized that a molecule that mimics the BST-2 extracellular domain will efficiently block BST-2-mediated breast cancer cell to cell interaction. Thus, we developed a BST-2-based small peptide (B49) that specifically binds to the BST-2 extracellular domain. The effect of B49 in preventing cancer cell adhesion and inhibiting tumor growth has been documented in a patent filling by The University of Iowa Research Foundation. The patent WO2017/011375 not only provides information on B49 composition but also provides methods for using B49 to inhibit cancer cell adhesion and tumor growth. This manuscript aims to provide detailed evidence on the effect of B49 and the first analog of B49 (B49Mod1) on homotypic and heterotypic cancer cell adhesion and growth in 2D and 3D experimental models, as well as in a mouse model of breast cancer.

## Results

### B49 targets BST-2 and inhibits BST-2-mediated homotypic cell adhesion

**Figure 1:**
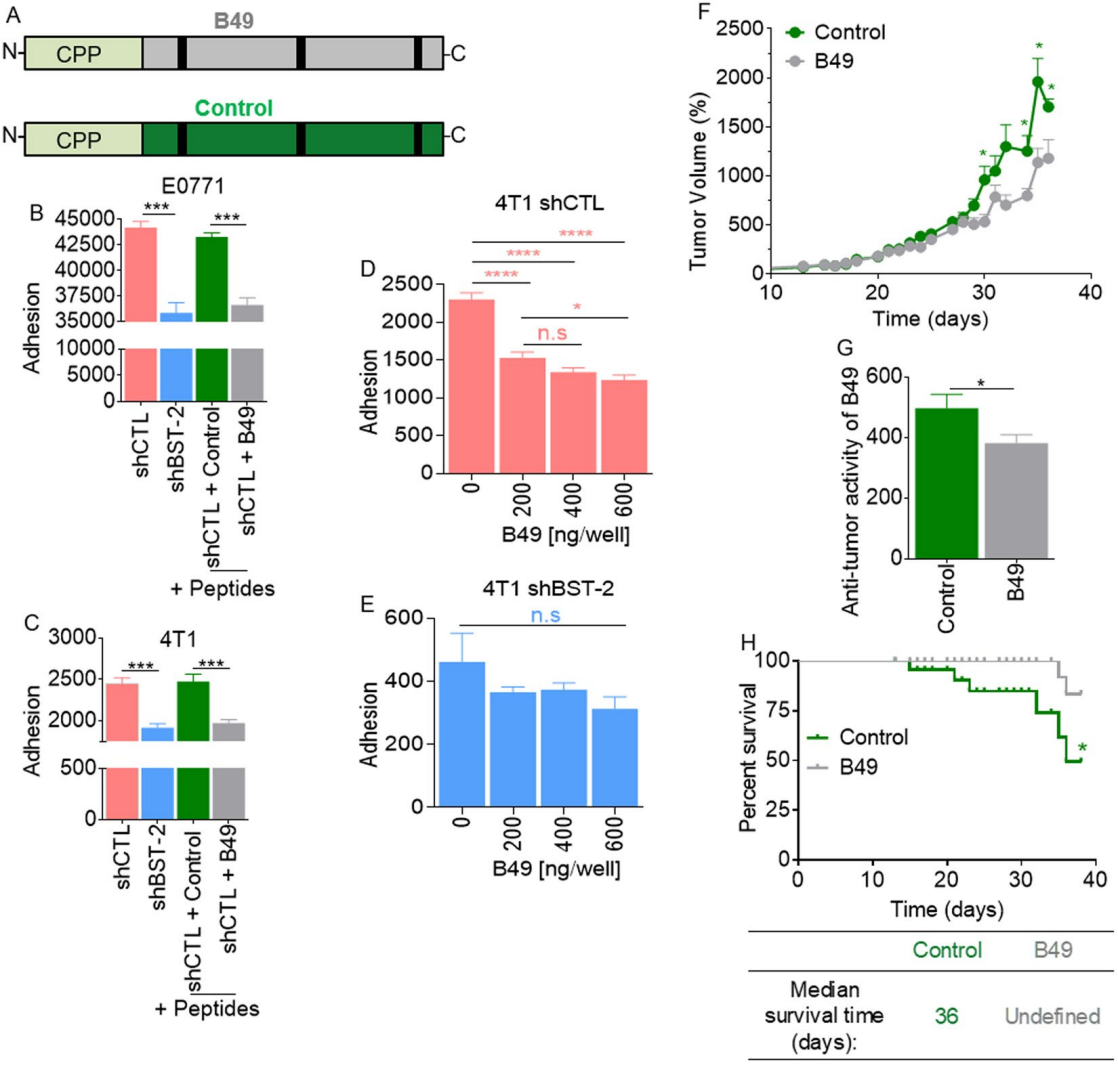
B49 targets BST-2 in cancer cells to prevent cell adhesion *in vitro* and tumor growth *in vivo*. (**A**) Cartoon depiction of a non-targeting control peptide (Control, green) and a BST-2-targeting peptide (B49, gray). On the N-terminus, both peptides contain penetratin (CPP). (**B**) Adherence of E0771 shControl (shCTL, pink) and E0771 shBST-2 (blue) breast cancer cells to E0771 shControl monolayers pre-treated with vehicle (1:8 (v/v) acetonitrile:water) or with 200 ng/well of the Control peptide (green) or B49 peptide (gray). (**C**) Adherence of 4T1 shControl (shCTL, pink) and 4T1 shBST-2 (blue) breast cancer cells to 4T1 shControl monolayers pre-treated with vehicle (1:8 (v/v) acetonitrile:water) or with 200 ng/well of the Control peptide (green) or B49 peptide (gray). (**D**,**E**) Adherence of (**D**) 4T1 shCTL cells or (**E**) 4T1 shBST-2 cells to 4T1 shControl monolayers pre-treated with 0, 200, 400 or 600 ng/well of B49. (**F**) Tumor volume over time computed as TV = 0.5 (length × width^2^) from mice (n = 15) bearing 4T1 tumors treated with a control peptide (green) or with B49 (gray). Once tumors reached 100 mm^3^, mice were treated intratumorally every three days with 0.3 μg/μl of peptide at 0.33 μl/mm^3^ tumor. Y-axis is represented as a percentage were initial tumor volume prior to peptide treatments was set to 100%. Tumor volume (%) = (Tumor volume on day X * 100)/100 mm^3^). (**G**) Anti-tumor activity of Control and B49 peptide calculated by averaging the tumor volumes from day 10 to day 35 (experimental end-point). (**H**) Kaplan–Meier survival plot of mice bearing 4T1 tumors treated with Control or B49 peptides over the course of 35 days. Median survival time is shown for each group. Error bars correspond to SEM. Significance was taken at *P < 0.05, ***P < 0.001 and ****P < 0.0001. ns = not significant.

As the BST-2 extracellular domain is critical for BST-2-mediated tumorigenesis, including mediating interactions between cancer cells and other components of the tumor microenvironment, promoting cancer cell survival through activation of the BST-2/GRB2/ERK/BIM/Cas3 signaling pathway, and promoting tumor growth and survival^6^, we designed B49 that binds to the BST-2 extracellular domain and a non-binding Control peptide (Fig. 1A) and then assessed the efficacy of B49 in blocking cancer cell adhesion. We show that B49 blocks homotypic adhesion of murine E0771 (Fig. 1B) or 4T1 (Fig. 1C) cells with the same efficacy as suppression of BST-2 in these cells. A subtle dose dependent effect of B49 was observed from 200 ng to 600 ng, although use of a higher dose does not result in greater net gain in adhesion inhibition (Fig. 1D). Interestingly, B49 did not inhibit adhesion in shBST-2 cells (Fig. 1E). The figures in 1D and 1E indicate that B49 functions in a BST-2-dependent manner and thus, BST-2 is a specific target for B49.

**Figure 2:**
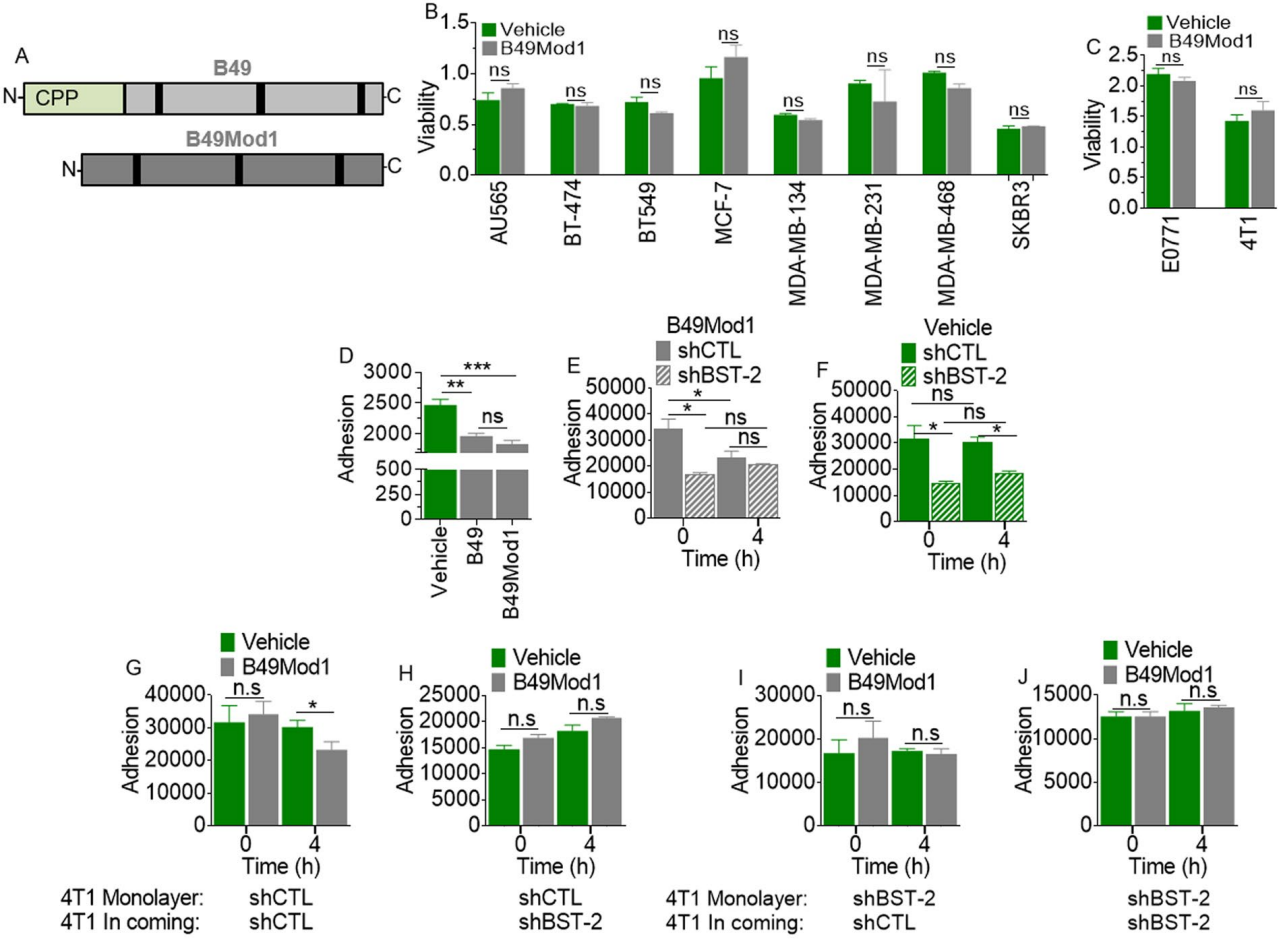
B49Mod1 completely retains the anti-adhesion function of B49 without affecting cell viability. (**A**) Cartoon depiction a BST-2-targeting peptide (B49) and a smaller B49 analog that lacks penetratin at the N-terminus (B49Mod1) and is more stable as defined by half-life in silico analyses. (**B**,**C**) Cellular viability of several (**B**) human and (**C**) mouse breast cancer cell lines following a 24-hour treatment with vehicle (1:8 (v/v) acetonitrile:water) or with 200 ng/well of B49Mod1. (**D**) Adherence of E0771 shControl (shCTL) cells to E0771 shControl monolayers that were pre-treated with Vehicle, B49, or B49Mod1 for 4 hours at 200 ng/well before addition of incoming cells. (**E**,**F**) Adherence of PKH67-labeled E0771 shControl or shBST-2 cells to E0771 shControl monolayers that were pre-treated with (**E**) B49Mod1 at 200 ng/well or (**F**) with an equivalent volume of Vehicle for 4 hours. (**G**,**H**) Adherence of 4T1 (**G**) shControl or (**H**) shBST-2 cells to 4T1 shControl monolayers that were pre-treated with Vehicle (green) or B49Mod1 (gray) at 200 ng/well for 4 hours. (**I**,**J**) Adherence of 4T1 (**I**) shControl or (**J**) shBST-2 cells to 4T1 shBST-2 monolayers that were pre-treated with Vehicle (green) or B49Mod1 (gray) at 200 ng/well for 4 hours. Error bars correspond to SEM. Significance was taken at *P < 0.05, **P < 0.01 and ***P < 0.001. ns = not significant.

### Efficacy of B49 treatment in a syngeneic mouse model of breast cancer

Here, we assessed the therapeutic potential of B49 by evaluating the efficacy of the peptide in inhibiting mammary tumor growth. We used the 4T1/Balb/c syngeneic breast cancer model and injected 4T1 cells into the mammary fatpad as previously described^6,11^. B49 was not toxic and did not affect the energy balance of mice since we did not observe any difference in food and water consumption between Control- or B49-treated mice. Tumor volume was measured from the first day of treatment and the relative tumor volume (RTV) was calculated using the following formula: RTV = (Vx/V1), where Vx is the tumor volume on day X and V1 is the tumor volume at initiation of the treatment. As shown in Fig. 1F, B49 treatment reduced tumor volume over control treatment particularly towards the end of the treatment. We observed that B49 induced a modest but significant tumor growth inhibition (35%). Analysis of average tumor inhibition for the length of treatment reveals that B49 had a significant tumor inhibitory effect over control peptides (Fig. 1G). Kaplan Meier survival analysis further reveals that by the experimental end point, 51% of control-treated mice have died while only about 17% of B49-treated mice died (Fig. 1H), suggesting that mice treated with B49 had a significant survival advantage over control-treated mice.

### Development and characterization of a first generation B49 analog (B49Mod1)

Since B49 binds to the BST-2 extracellular domain, we hypothesized that internalization of B49 is not necessary for its function. This hypothesis was tested by synthesizing an analog of B49 herein named B49Mod1 by removing the cell penetrating peptide (CPP) and adding two amino acids to each of the C and N terminus (Fig. 2A). Evaluation of sensitivity of various human breast cancer cell lines to B49Mod1 was performed by comparing viability of cells of interest to vehicle control following exposure to B49Mod1. Cell viability analyzed by MTT assay at 24 h show that treatment with B49Mod1 is not toxic to cells (Fig. 2B). A similar observation was made in murine cell lines—E0771 and 4T1 cells where B49Mod1 does not have effect on cell viability (Fig. 2C). As a whole, B49Mod1 showed no cytotoxic effects on all cell lines evaluated, suggesting that cells tolerate B49Mod1.

Comparison of anti-adhesion activity of B49Mod1 to parental B49 shows that removal of CPP does not have effect on the ability of B49Mod1 to inhibit adhesion (Fig. 2D), indicating that peptide internalization is not necessary for its function. Although inhibition efficacy of parental B49 and B49Mod1 are not statistically different, B49Mod1 is more inhibitory than parental B49 when compared to vehicle (Fig. 2D).

To more closely examine the effect of B49Mod1, we compared B49Mod1-mediated inhibition of cancer cell adhesion with the inhibition obtained with shRNA-mediated BST-2 suppression. We observed significant inhibition of cancer cell adhesion by B49Mod1 at 4 h (Fig. 2E) that paralleled inhibition observed when BST-2 was shRNA-suppressed in these cells (Fig. 2F). Similar to parental B49, the B49Mod1 analog inhibits adhesion in a BST-2 dependent manner because the peptide does not have effect on the adhesion of BST-2-suppressed shBST-2 cells (Fig. 2E). Furthermore, treatment of BST-2-expressing shCTL monolayer with B49Mod1 inhibits adhesion of incoming BST-2-expressing cells (Fig. 2G) but does not have effect on incoming BST-2-suppressed shBST-2 cells (Fig. 2H). Interestingly, when a BST-2-suppressed shBST-2 monolayer is treated with B49Mod1, neither BST-2-expressing shCTL (Fig. 2I) nor BST-2-suppressed shBST-2 (Fig. 2J) incoming cells adhere efficiently to the B49Mod1-treated monolayer. These results indicate that B49Mod1 functions in a BST-2-dependent manner to prevent cancer cell to cancer cell interactions.

### B49Mod1 inhibits heterotypic adhesion between cancer cells and components of the tumor microenvironment

**Figure 3:**
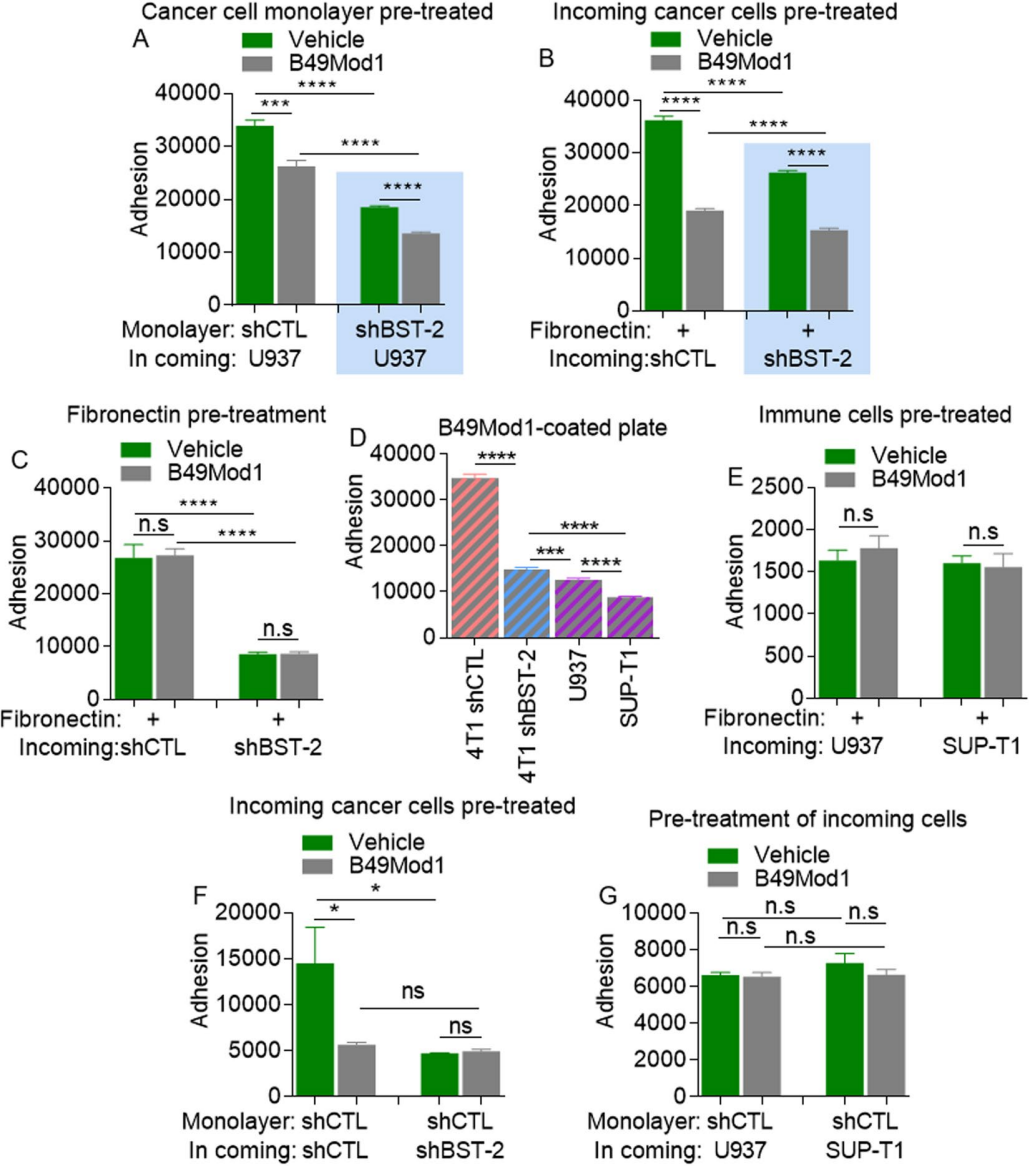
B49Mod1 inhibits homotypic and heterotypic adhesion of cancer cells specifically. (**A**) Adherence of U937 monocytes to 4T1 shControl (shCTL, no background) or shBST-2 (blue background) monolayers that were pre-treated with Vehicle or B49Mod1 for 4 hours at 200 ng/well before addition of incoming cells. (**B**) Adherence of 4T1 shControl (no background) or shBST-2 (blue background) cancer cells to fibronectin-coated plates. Incoming cancer cells were pre-treated with vehicle or B49Mod1 for 2 hours prior to addition to fibronectin-coated plates. (**C**) Adherence of 4T1 shControl (no background) or shBST-2 (blue background) cancer cells to fibronectin-coated plates that were pre-treated with 200 ng/well of B49Mod1 for 4 hours and washed once before addition of incoming 4T1 cancer cells. (**D**) Adherence of 4T1 shControl, 4T1 shBST-2, U937 monocytes or SUP-T1 T cells to B49Mod1-coated plates. (**E**) Adherence of U937 monocytes and SUP-T1 T cells to fibronectin-coated plates. Incoming immune cells were pre-treated with vehicle or B49Mod1 for 2 hours prior to addition to fibronectin-coated plates. (**F**) Adherence of 4T1 shControl (no background) or shBST-2 (blue background) cancer cells to 4T1 shControl monolayers. In coming cancer cells were pre-treated with vehicle or B49Mod1 for 2 hours prior to addition to 4T1 shControl monolayers. (**G**) Adherence of U937 monocytes or SUP-T1 T cells to 4T1 shControl monolayers. In coming immune cells were pre-treated with vehicle or B49Mod1 for 2 hours prior to addition to 4T1 shControl monolayers. Error bars correspond to SEM. Significance was taken at *P < 0.05, ***P < 0.001 and ****P < 0.0001. ns = not significant.

We have previously shown that BST-2 mediates heterotypic adhesion between cancer cells and components of the tumor microenvironment, including immune cells and extracellular matrix substrates (collagen and fibronectin)^6^. To determine if B49Mod1 inhibits BST-2-mediated formation of heterotypic cell-cell or cell-matrix adhesions, we used a co-culture system in which monolayers of unlabeled, B49Mod1 pre-treated shCTL or shBST-2 cells were co-cultured with PKH67Green-labeled incoming monocytes to distinguish between the two cell types. Analysis of adhesion shows that heterotypic shCTL:U937 or shBST-2:U937 adhesion was significantly blocked by B49Mod1(Fig. 3A). This result suggests that B49Mod1 is effective in blocking both BST-2-mediated and non-BST-2-mediated immune cell adhesion to cancer cells. The effect of B49Mod1 on immune cell to cancer cell adhesion is partly BST-2-independent since adhesion of monocytes to shBST-2 was also inhibited by the peptide (Fig. 3A, shaded area).

**Figure 4:**
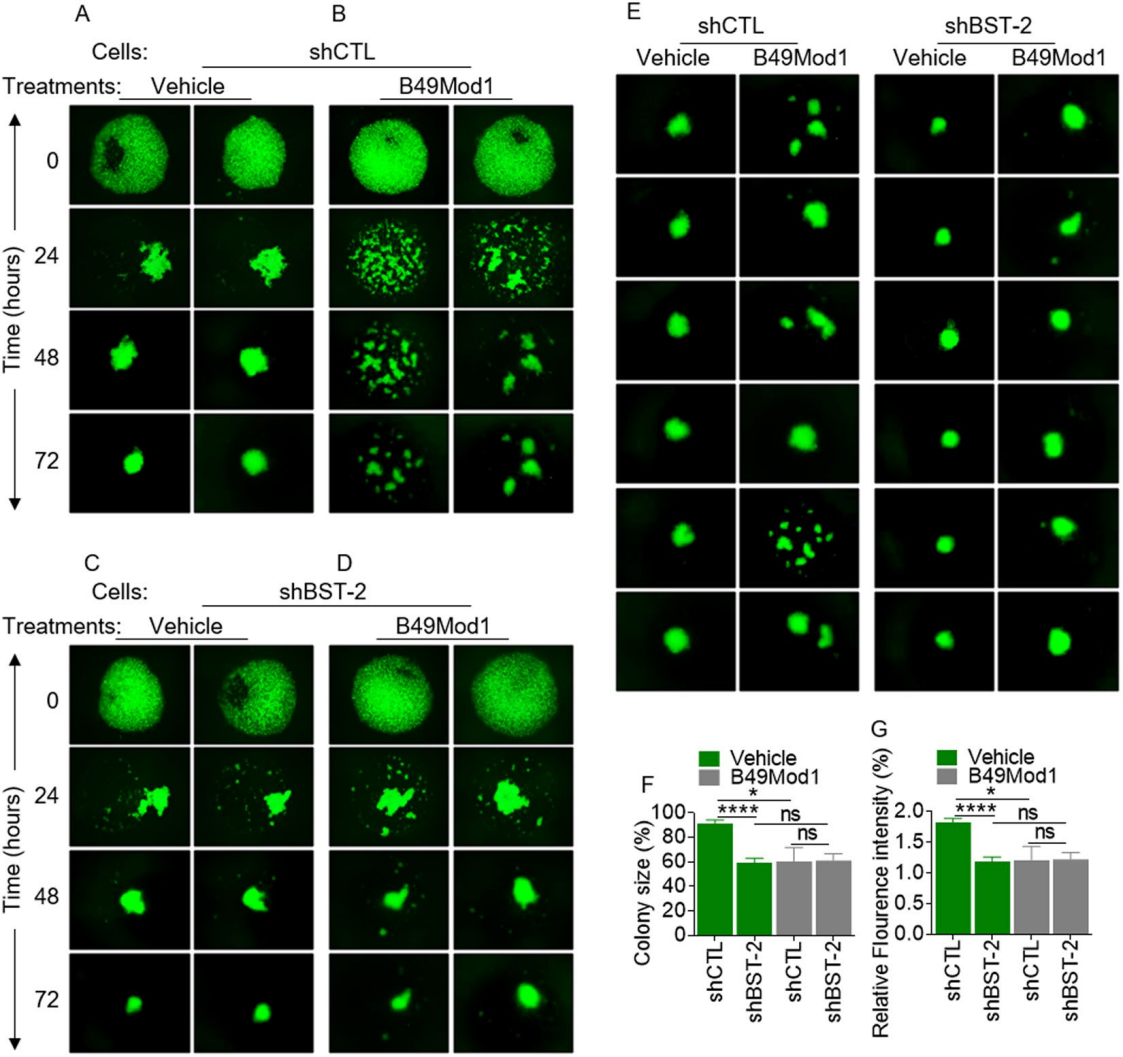
B49Mod1 impedes spheroid-formation of BST-2-expressing breast cancer cells. (**A**,**B**) Representative 4× images of PKH67-labeled 4T1 shControl (shCTL) spheroids formed on Poly-HEMA-coated plates at 0, 24, 48, and 72 hours. 4T1 shControl cells were treated once with (**A**) Vehicle or with (**B**) 500 ng/well of B49Mod1. (**C**,**D**) Representative 4× images of PKH67-labeled 4T1 shBST-2 spheroids formed on Poly-HEMA-coated plates at 0, 24, 48, and 72 hours. 4T1 shBST-2 cells were treated once with (**C**) Vehicle or with (**D**) 500 ng/well of B49Mod1. (**E**) 4× images of spheroids formed at 72 hours on Poly-HEMA-coated plates by PKH67-labeled shControl (left) and shBST-2 (right) 4T1 cells treated with Vehicle or B49Mod1 as described previously. (**F**) Colony size of 4T1 shControl (shCTL) and shBST-2 cells treated with Vehicle (green) or B49Mod1 (gray) at 72 hours post-treatment on Poly-HEMA-coated plates. Colony size is represented as a percent relative to the colony size of vehicle-treated shControl 4T1 cells which was set to 100%. In the case of B49Mod1-treated shCTL cells, the biggest fragment in the field of view was measured. (**G**) Relative fluorescence intensity of 4T1 shControl (shCTL) and shBST-2 cells treated with Vehicle (green) or B49Mod1 (gray) at 72 hours post-treatment on Poly-HEMA-coated plates. Relative fluorescence intensity was measured using ImageJ. In the case of B49Mod1-treated shCTL cells, the biggest fragment in the field of view was selected and fluorescence measured. Error bars correspond to SEM. Significance was taken at *P < 0.05 and ****P < 0.0001. ns = not significant.

Given the BST-2-independent effect of B49Mod1 in blocking immune cell to cancer adhesion, we developed a system to evaluate the effect of B49Mod1 on other adhesion molecules that may mediate cell: matrix interactions in the absence of BST-2. We pre-labeled shCTL and shBST-2 cells with PKH dye and pre-treated cells with vehicle or B49Mod1. Cells were then seeded in wells that have been pre-coated with fibronectin. Analysis of adhesion reveals that B49Mod1 blocks adhesion of cancer cells to fibronectin-coated plates (Fig. 3B). This effect was independent of BST-2 because B49Mod1 blocks the binding of shBST-2 to fibronectin-coated plates (Fig. 3B, shaded area). The observed blockade of the interaction between cancer cells and fibronectin was not due to B49Mod1 blocking fibronectin since B49Mod1 does not block adhesion of shCTL or shBST-2 cancer cells to fibronectin-coated B49Mod1-treated plate (Fig. 3C). Indeed, cancer cells efficiently bind to B49Mod1-coated plates in a BST-2-dependent manner (Fig. 3D). Since B49Mod1 blocks cancer cell binding to fibronectin and B49Mod1 does not bind to fibronectin, we examined the effect of the peptide in blocking immune cell binding to fibronectin. We pre-treated PKH67Green-labelled monocytes (U937) and T cells (SUP-T1) with B49Mod1 and seeded cells on to fibronectin-coated plates. Rate of cell adherence was not different between vehicle and B49Mod1 treatment (Fig. 3E).

Next, we assessed the adhesion of B49Mod1-treated incoming cancer or immune cells on cancer cell monolayers. In this condition, B49Mod1 efficiently blocked adhesion of incoming-treated cancer cells to untreated cancer cell monolayer in a BST-2-dependent manner (Fig. 3F). At variance, B49Mod1 did not block adhesion of B49Mod1 pre-treated immune cells to untreated cancer cell monolayer (Fig. 3G), despite blockade of the adhesion of immune cells to B49Mod1 pre-treated cancer cell monolayer (Fig. 3A). Together, these results suggest that i) B49Mod1 inhibits adhesion of BST-2-expressing and BST-2-suppressed cancer cells but not immune cells to ECM substrates, ii) immune cells whether incoming or resident are not responsive to B49Mod1-mediated inhibition of adhesion, and iii) monolayer cells treated with B49Mod1 may not provide a permissive niche for incoming BST-2-expressing tumor or immune cells.

### B49Mod1 disrupts homotypic breast cancer cell aggregation, decreases rate of spheroid formation, and alters spheroid morphology

As shown in Fig. 4A,B, the 3D structures formed by 4T1 shCTL cells treated with vehicle differed from B49Mod1-treated structures. Under both vehicle and B49Mod1 conditions, cancer cells are capable of clustering/aggregation at time point 0 when cell spheroids/aggregates are grown on Poly-HEMA. However, at 24 h, vehicle-treated cells form tight/compact, multicellular aggregates (Fig. 4A) while B49Mod1-treated cells remained as loosely-associated aggregates with intercellular spacing (Fig. 4B). By 72 h, vehicle-treated cells progressively formed circular compact spheroids (Fig. 4A) and B49Mod1-treated cells still contained multiple irregular-shaped spheroids separated by large intercellular spaces (Fig. 4B). The time required to form “true spheroids” (defined as tight/compact, circular structures) is between 48 h and 72 h in vehicle-treated cells (Fig. 4A). The differences in spheroid structure between vehicle-treated and B49Mod1-treated cells remained up to 72 h because spheroids treated with vehicle remained compact and circular while B49Mod1-treated spheroids remained fragmented and irregular with both concave and convex-shaped structures. Spheroid size was not determined since B49Mod1 disrupted spheroidization resulting in fragmentation of the normally well-circumscribed spheroids (compare Fig. 4A and B, 72 h).

Specificity of B49Mod1 in disruption of BST-2-mediated spheroidization was further demonstrated using shBST-2 cells in which BST-2 has been suppressed^6,11^. Compared to BST-2-expressing (shCTL) spheroids (Fig. 4A), shBST-2 spheroids are fragmented at 24 h and smaller in size up to 72 h (Fig. 4C). Unlike the effect of B49Mod1 on BST-2-expressing shCTL cells (Fig. 4B), the peptide does not have further spheroid disruptive effect on BST-2-suppressed shBST-2 cells (Fig. 4D). On the average, spheroids formed by shCTL cells are larger in size compared to shBST-2 spheroids (Fig. 4E,F). B49Mod1 disrupts the process of spheroidization that is mediated by BST-2 and has no further effect on spheroidization of BST-2-suppressed cells (Fig. 4E,F). Since the cells were labeled with PKH67Green, we used fluorescence intensity as a second readout for spheroid size. We found that B49Mod1 decreased fluorescence of shCTL cells to the level of shBST-2 cells without further decrease in shBST-2 cells (Fig. 4G). These data indicate that BST-2 is necessary for breast cancer spheroid growth but B49Mod1 disrupts BST-2-mediated breast cancer cell spheroidization.

### Heterotypic co-culture spheroidization is inhibited by B49Mod1

**Figure 5:**
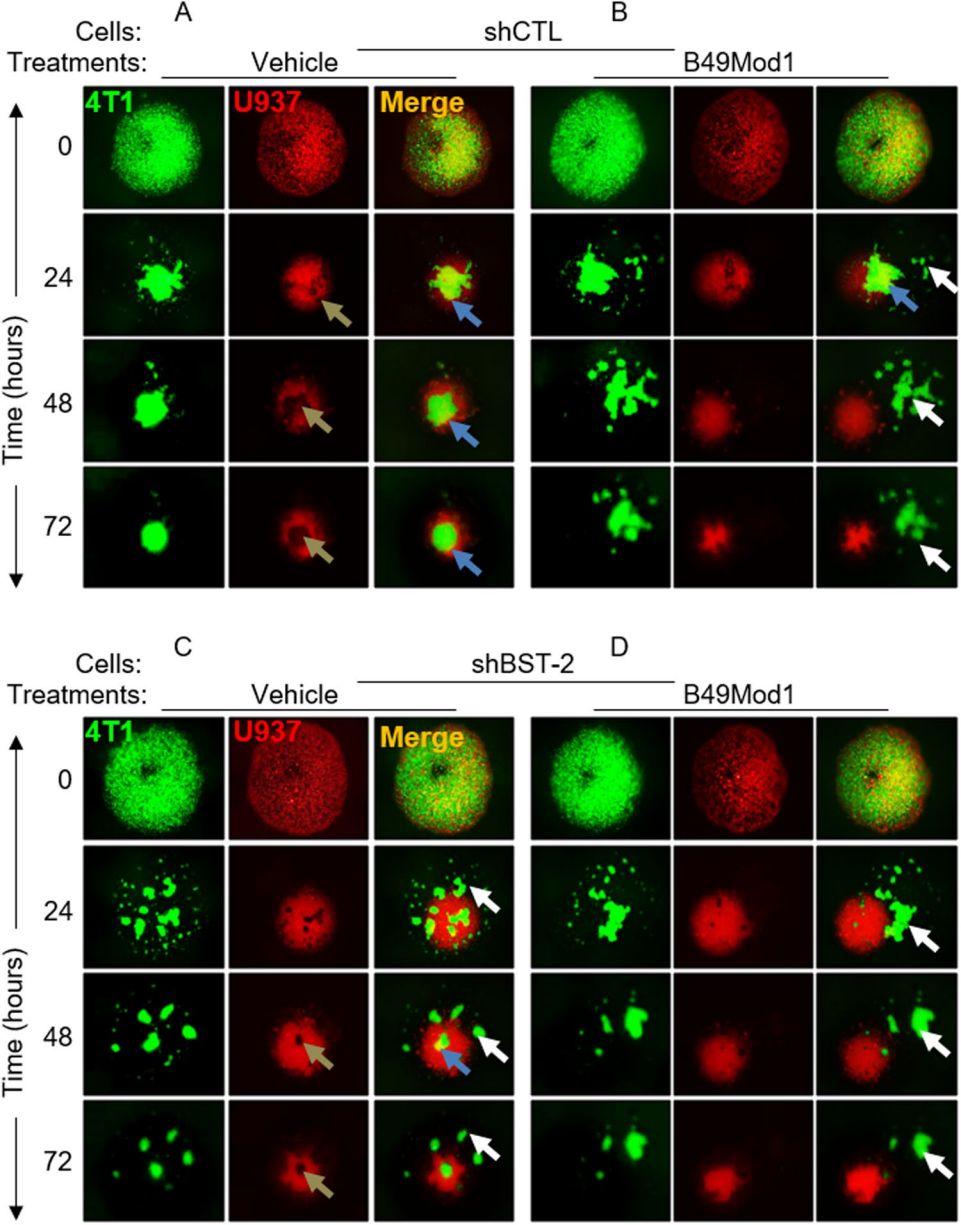
B49Mod1 blocks the interaction of monocytes with cancer cells in suspension. (**A**,**B**) Representative 4× images of PKH67Green-labeled 4T1 shControl (shCTL) and PKH26Red-labeled U937 spheroids formed on Poly-HEMA-coated plates at 0, 24, 48, and 72 hours. 4T1 shControl cells and U937 cells were treated once with (**A**) Vehicle or with (**B**) 500 ng/well of B49Mod1. Gold arrows represent the location of green-labeled cancer cells within the cluster of U937 cells while blue arrows depict green-labeled cancer cells in contact with U937 cells. White arrows depict fragments of green-labeled cancer cells that are not interacting with U937 cells. (**C**,**D**) Representative 4× images of PKH67Green-labeled 4T1 shBST-2 and PKH26Red-labeled U937 spheroids formed on Poly-HEMA-coated plates at 0, 24, 48, and 72 hours. 4T1 shBST-2 cells and U937 cells were treated once with (**C**) Vehicle or with (**D**) 500 ng/well of B49Mod1. Gold arrows represent the location of green-labeled cancer cells within the cluster of U937 cells while the blue arrow depicts green-labeled cancer cells in contact with U937 cells. White arrows depict fragments of green-labeled cancer cells that are not interacting with U937 cells.

Since homotypic cancer cell spheroidization is disrupted by B49Mod1, we performed mixed cancer cells and monocytes spheroid coculture to study the potential effect of tumor microenvironment mediated by the immune cells on B49Mod1 efficacy. The monocytes (red) and cancer cells (green) colocalized (orange) shortly after (0 h) plating on Poly-HEMA (Fig. 5A–D). The introduction of monocytes did not change the dynamics of spheroidization observed in homotypic spheroids (Fig. 4) when considering spheroid size and formation time. In the vehicle coculture, cancer cell:monocyte interaction was maintained throughout the culture period (Fig. 5A, blue arrow heads), with tight/compact spheroids forming by 72 h. Cancer cells are shown surrounded by immune cells in a close interaction (Fig. 5A, gold arrow heads). However, B49Mod1 was effective in disrupting this interaction since minimal contact/colocalization was observed from 24 h onwards (Fig. 5B, blue arrow head). In addition, B49Mod1 fragmented cancer cell spheroids even in the presence of monocytes (Fig. 5B, white arrow heads). For specificity, we evaluated the effect of B49Mod1 on heterotypic spheroidization between shBST-2 and monocytes. Significant spheroid fragmentation was observed (Fig. 5C, white arrow heads) with minimal cancer cell and monocytes interaction (Fig. 5C, blue and gold arrow heads). Similar to homotypic spheroidization, B49Mod1 does not have significant spheroidization disruptive effect on shBST-2 cells (Fig. 5D). These results suggest that B49Mod1 has the potential to disrupt cancer cell interaction with immune cells in the tumor microenvironment.

### Cell expansion decreases with B49Mod1 treatment in 3D clonogenic culture model

**Figure 6:**
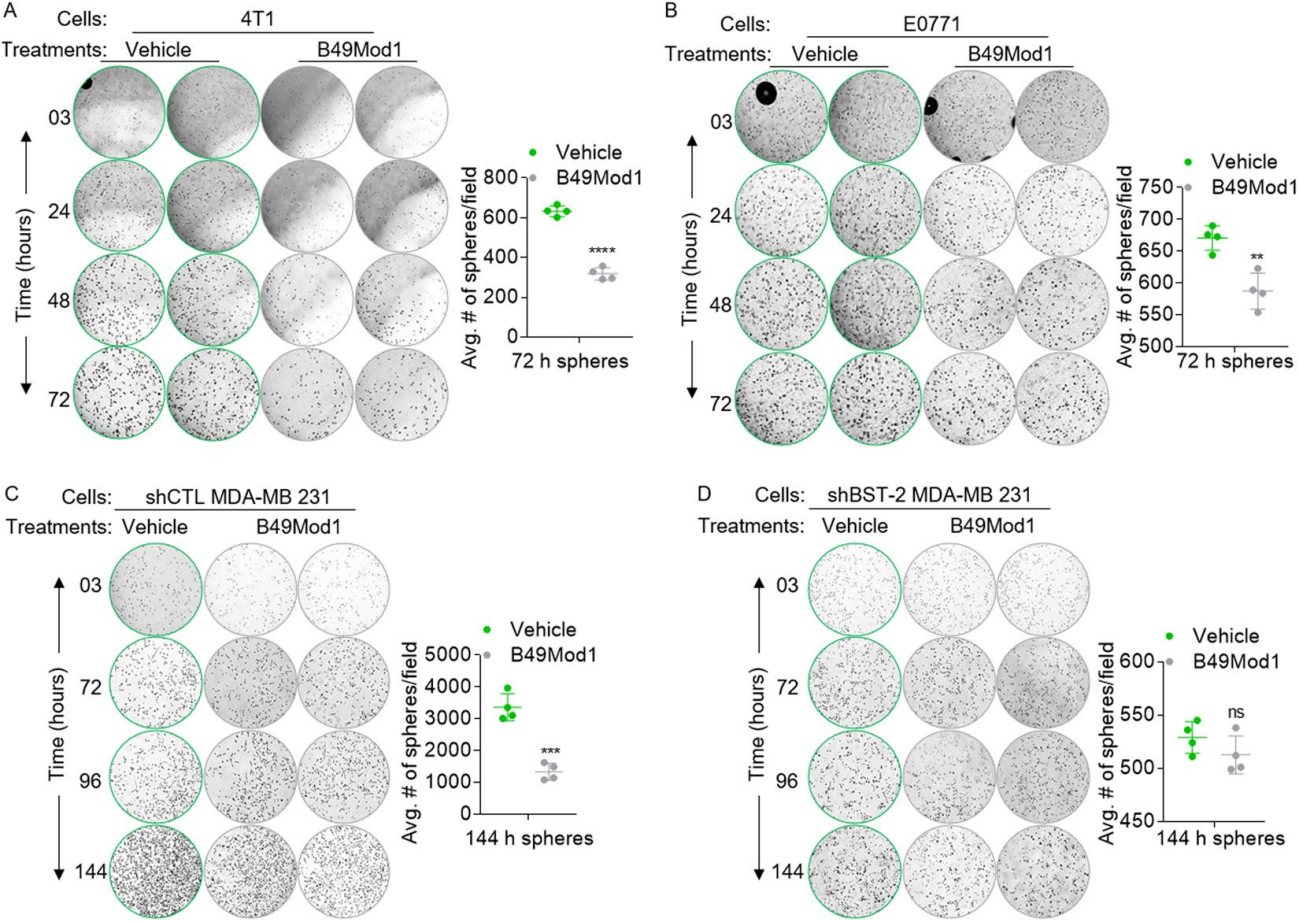
Time-dependent effect of B49Mod1 on cancer cell expansion in clonogenic 3D cultures. (**A**,**B**) Representative 4× images and quantification of spheres formed by (**A**) 4T1 cells, (**B**) E0771, (**C**) shCTL MDA-MB 231 and (**D**) shBST-2 MDA-MB 231 cells embedded in VitroGel 3D and treated with Vehicle or B49Mod1 (500 ng/well). Graphs show average number of spheres per field. Error bars correspond to SEM for sphere quantification. Significance was taken at **P < 0.01, ***P < 0.001 and ****P < 0.0001. ns = not significant.

The substantial disruption of spheroidization of cancer cells by B49Mod1 suggests that the peptide may also inhibit adhesion-independent cell growth. Hence, we performed a series of 3D clonogenic spheroid assays in which cells were embedded within matrix over time. The results show that B49Mod1 inhibits cell growth as indicated by reduced colony density (images) and colony numbers (graphs) in Fig. 6A–C. The effect of B49Mod1 is species and cell type independent since both murine (4T1, E0771) and human (MDA-MB 231) cells were responsive to B49Mod1-mediated effect. Of note, the growth inhibition was absent in MDA-MB 231 cells in which BST-2 expression has been suppressed (Fig. 6D) with BST-2-targeting shRNA^23^. These results show that even in 3D-embedment culture conditions, B49Mod1 inhibits cancer cell growth in a BST-2-dependent manner.

### B49Mod1 inhibits BST-2-mediated anchorage-independent growth (AIG)

**Figure 7:**
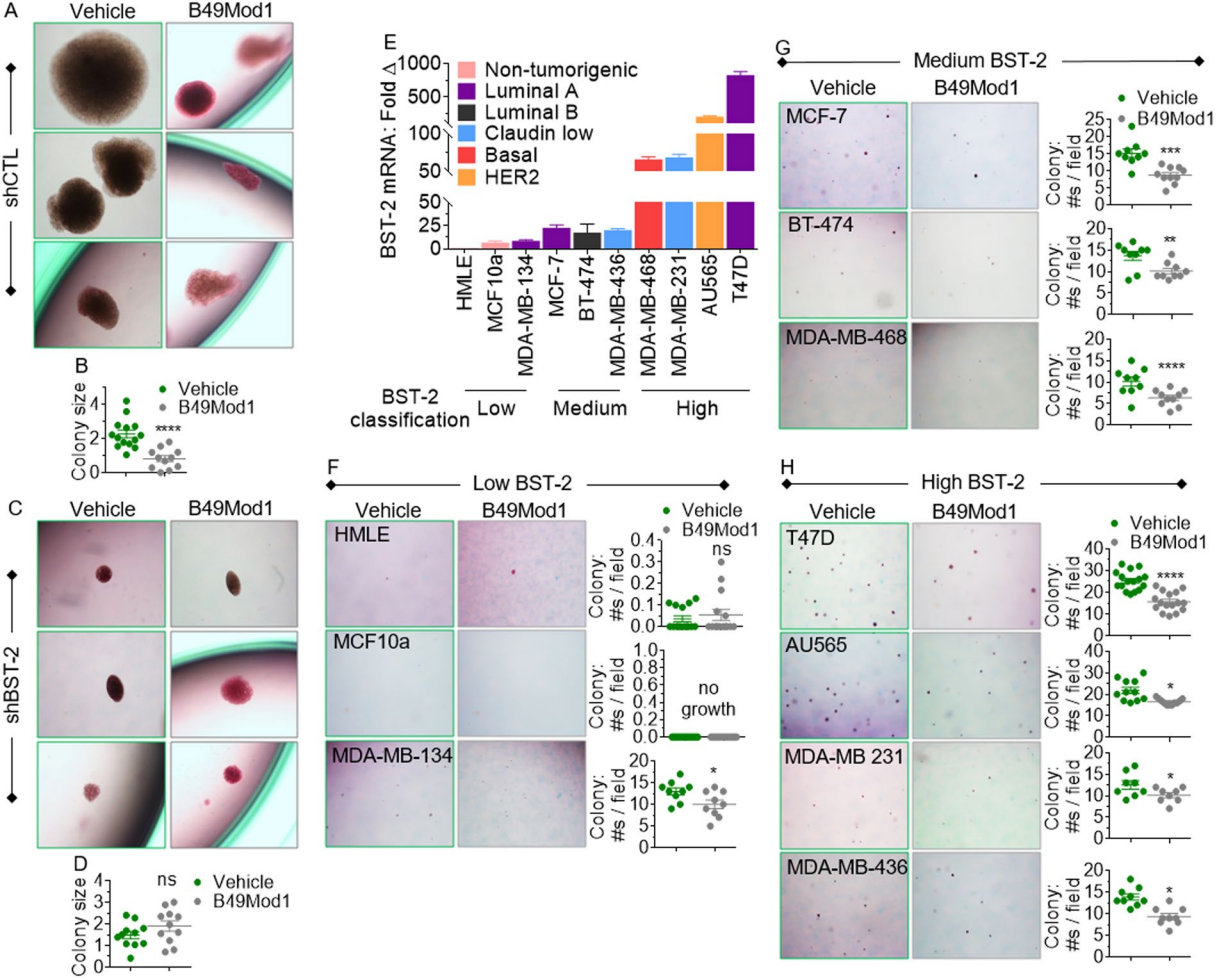
B49Mod1 impedes anchorage-independent growth of cancer cells irrespective of breast cancer subtype classification. (**A**–**D**) Representative 4× images (**A**,**C**) and quantification (**B**,**D**) of colonies formed by E0771 cells expressing a non-targeting shRNA (shControl, shCTL) or a BST-2-targeting shRNA (shBST-2) and treated with Vehicle or B49Mod1 (500 ng/well) every 3 days for a 30-day anchorage-independent growth assay. (**E**) Human BST-2 RNA levels in two normal mammary epithelial cells (HMLE and MCF-10A) and several human breast cancer cell lines classified according to their BST-2 levels. BST-2 classification is based on relative RNA fold change; low BST-2 = 1–10; medium BST-2 = 10–30, and high BST-2 = 30–1000. GAPDH was used for normalization of RT-qPCR data. (F-H) Representative 4× images and quantification of colonies formed by (**F**) low BST-2-expressing (**G**) medium BST-2-expressing, and (**H**) high BST-2-expressing human breast cancer cells that were treated with Vehicle or B49Mod1 (500 ng/well) every 3 days. Error bars correspond to standard deviation for RT-qPCR data and to SEM for colony quantification data. Significance was taken at *P < 0.05, **P < 0.01, ***P < 0.001 and ****P < 0.0001. ns = not significant.

Since B49Mod1 inhibits adhesion-independent cell growth, we used a different assay to evaluate the effect of B49Mod1 in long term cell growth. For this purpose, we studied the effect of B49Mod1 in anchorage-independent colony formation in soft agar. We found significant difference in anchorage-independent growth in the presence of B49Mod1 (Fig. 7A,B), where colony size was reduced by B49Mod1 compared to vehicle control. The effect of B49Mod1 is dependent on BST-2 since treatment of BST-2-suppressed shBST-2 cells with B49Mod1 produced colonies of similar sizes (Fig. 7C,D).

### B49Mod1 inhibits AIG in different subtypes of human breast cancer cells

Similar to the effects of B49Mod1 on murine cell lines, a number of human non-tumorigenic (HMLE and MCF10a) and tumorigenic cell lines representing the 5 breast cancer subtypes (luminal A, luminal B, claudin low, basal, and HER2) with varying levels of BST-2 (low, medium, and high) mRNA (Fig. 7E) were analyzed for colony formation in the presence of B49Mod1 (Fig. 7F–H). B49Mod1 does not have an effect on anchorage-independent growth of low BST-2 expressing non-tumorigenic HMLE and MCF10a cells that form little or no colonies, but inhibited growth of MDA-MB 134 luminal A breast cancer cell colonies (Fig. 7F) despite low levels of BST-2 (Fig. 7E). In addition, B49Mod1 inhibits the growth of other cell lines, including MCF-7 luminal A, BT-474 luminal B, and MDA-MB 464 basal subtypes (Fig. 7G) that express medium BST-2 (Fig. 7E). The inhibitory effect of B49Mod1 was also observed in high BST-2 expressing cell lines (Fig. 7E) representing various subtypes, such as T47D luminal A, AU565 HER2, MDA-MB 231 and MDA-MB 436 claudin low subtypes (Fig. 7H). These data indicate that B49Mod1 inhibits anchorage-independent growth of breast cancer cells regardless of the subtype or level of BST-2, but does not have effect on non-tumorigenic cell lines, suggesting a strong therapeutic potential against metastatic breast cancer.

### B49Mod1 disrupts cysteine-linked BST-2-mediated cell to cell interaction

**Figure 8:**
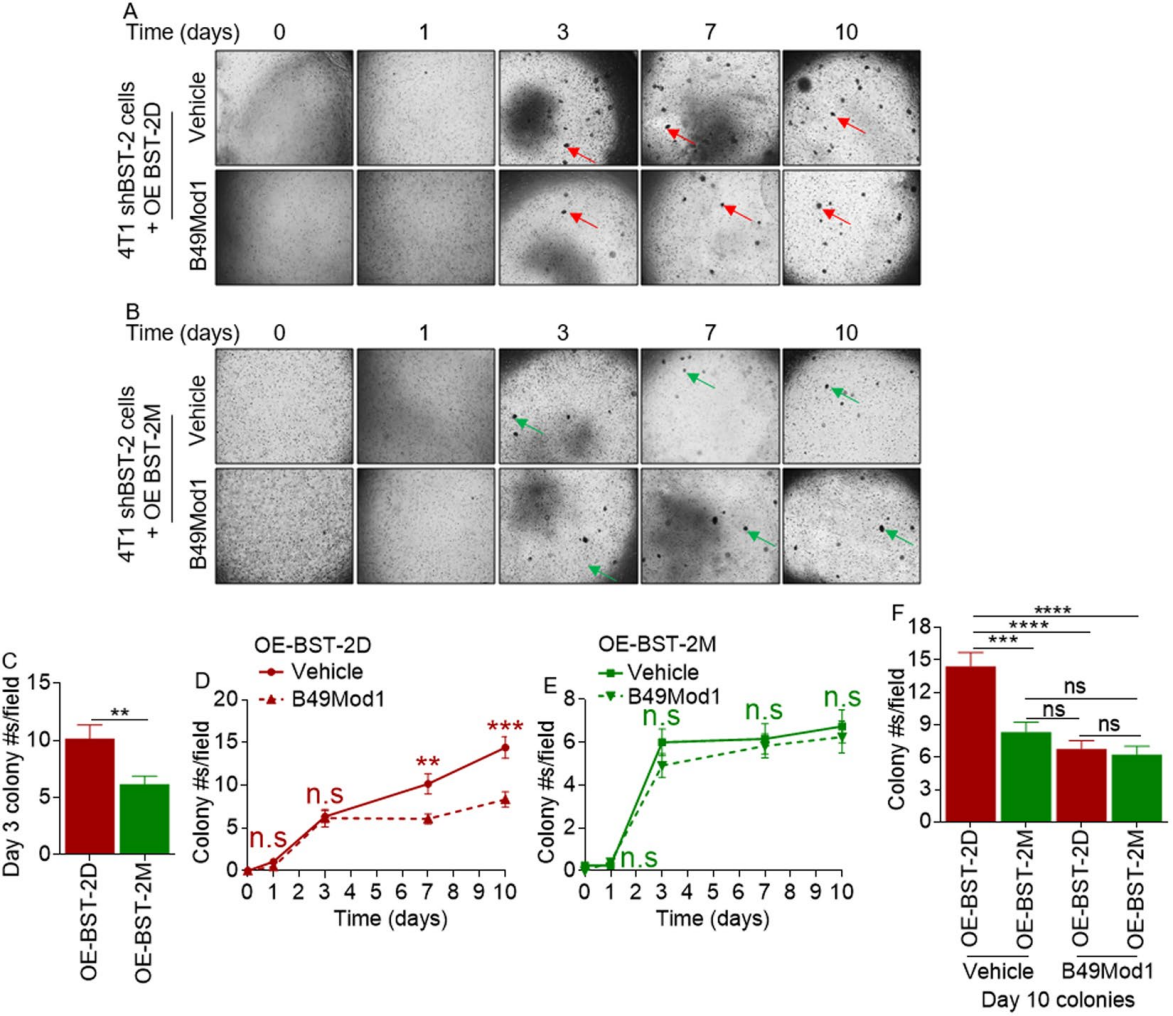
BST-2-dimerization is required for B49Mod1-mediated inhibition of cell growth. (**A**,**B**) Representative 4× images of colonies formed on VitroGel 3D by 4T1 shBST-2 cells overexpressing (**A**) a wild-type form of human BST-2 that is dimerization-competent (OE BST-2D) or (**B**) a dimerization-incompetent human BST-2 expressed as monomers (OE BST-2M) over a 10-day period. Cells were either treated with Vehicle or with 200 ng/well of B49Mod1. Peptide and media were replaced every two days until the end of the experiment. Red arrows depict OE BST-2D 4T1 colonies formed in suspension and green arrows depict smaller and fewer colonies formed by OE BST-2M 4T1 cells in suspension. (**C**–**F**) Quantification of colony numbers formed by OE BST-2D (red) and OE BST-2M (green) 4T1 cells at (**C**) day 3, (**D**,**E**) over a 10 day period or (**F**) at 10 days. Error bars correspond to SEM. Significance was taken at **P < 0.01, ***P < 0.001 and ****P < 0.0001. ns = not significant.

Given that B49Mod1 inhibits anchorage-independent growth of tumorigenic cells but does not have effect on shBST-2 cells attenuated for tumorigenesis (Figs 6A,D and 7A to D), we aimed to closely evaluate the structural requirement of BST-2 that support B49Mod1 function. We utilized previously reported 4T1 shBST-2 cell lines^6^ that overexpress two variants of BST-2, namely, dimerization-proficient BST-2 (OE-BST-2D) or dimerization-deficient BST-2 (OE-BST-2M)^6^ in a colony formation experiment using a hydrogel system— VitroGel 3D. We followed the kinetics of adhesion-independent growth over time and found that both OE-BST-2D and OE-BST-2M cells formed round colonies after approximately 3 days (Fig. 8A, red arrows and B, green arrows, top panels). As expected and as previously reported^6^, OE-BST-2D cells formed more colonies as early as day 3 compared to OE-BST-2M cells (Fig. 8A, red arrows and B, green arrows, top panels) and quantified in Figure 8C. Assessment of the effect of B49Mod1 on colony numbers show that OE-BST-2D cells treated with vehicle formed more colonies compared to their B49Mod1-treated counterparts (Fig. 8A,D). A similar analysis performed on OE-BST-2M cells treated with vehicle or B49Mod1 reveals that aside from OE-BST-2M mediated reduction in colony numbers, BST-2Mod1 does not have additional effect on OE-BST-2M colony numbers (Fig. 8B,E). Further tracking of colony numbers over time show that by day 10, B49Mod1 significantly decreased colony numbers of OE-BST-2D cells to the level observed in OE-BST-2M cells (Fig. 8A,B day 10 panel, and 7F). These data confirms that B49Mod1 disrupts BST-2-mediated cell to cell clustering and cell growth. In addition to the level of BST-2, the variant (BST-2D vs BST-2M) of BST-2 is important in B49Mod1 targeting because B49Mod1 is inactive in OE-BST-2M cells that predominantly express BST-2 monomers^6^.

## Discussion

The goal of this study is to characterize B49 (a 7644.83 molecular weight peptide) and its first analog B49Mod1 to better understand their applicability as a novel reagent for the study of cancer cell behavior and for oncology drug discovery. Our data show that B49 linked to a cell penetrating peptide (CPP) inhibits cancer cell adhesion and tumor growth in mice. Removal of the CPP and addition of new residues to B49 resulted in a bioactive analog B49Mod1, indicating that CPP-mediated peptide uptake is not necessary for B49 function.

While two-dimensional cell line studies were used to demonstrate the efficacy of B49Mod1 on homotypic and heterotypic cell to cell interaction, functional three-dimensional studies provided additional evidence for B49Mod1 efficacy on inhibition of cell clustering/spheroidization and anchorage-independent growth of tumor cells. As shown in our homotypic assay, B49Mod1 disrupts the complex network of 3D spheroid cell to cell contacts and subsequent formation of spheroid masses. These 3D studies provide insight into the potential for using B49Mod1 to reverse the malignant phenotype of highly aggressive breast cancer cells, irrespective of their subtype classification. In different culture formats, B49Mod1-treated cells exhibit very significant and interesting changes in behavior, including inhibition of adhesion, disruption of spheroidization, and inhibition of anchorage-independent growth. In some cell lines, colony size was inhibited and in others colony number was impaired. In the presence of B49Mod1, other parameters that can affect the potential metastatic behavior of cancer cells *in vivo* changed dramatically. For example, B49Mod1 inhibits cancer cell to matrix adhesion, homotypic and heterotypic adhesion, spheroidization and the development of spheroid masses that have been implicated in the formation of new metastases *in vivo*. These processes are enhanced by elevated BST-2^6,11^ and are key players in the pathogenesis of many cancers, but are inhibited by B49Mod1.

The significance of B49Mod1 in breast cancer becomes increasingly relevant with complex culture systems containing multiple cell types such as the mixture of cancer cells with monocytes (U937). In such systems, B49Mod1-mediated inhibition of adhesion and spheroid formation may be indicative of the immunomodulatory function of B49Mod1 since the peptide is able to inhibit the interaction between cancer cells and immune cells. The hypothesis that B49Mod1 will be effective in inhibiting tumor cell growth *in vivo* is supported by the demonstration that B49 decreased the rate of breast tumor growth by 35% in a syngeneic pre-clinical mouse model of breast cancer. These data are of interest since the syngeneic model takes into account the activities of the various host immune factors (soluble and cellular) as well as proteases that could deactivate the peptide or clear it from the system. It remains to be tested whether B49Mod1 has any immunogenic effects that help shape the tumor microenvironment one way or the other. Such effects are normally orchestrated by signaling pathways that connect extracellular signals to the nucleus, regulating expression of genes that directly or indirectly control cell adhesion and growth.

The mechanisms of action of B49 and its analog B49Mod1 are not known. However, we have shown in a cell-based mutagenesis study that replacing cysteine residues in the extracellular domain of BST-2 with alanine residues results in cells expressing BST-2 monomers^6^. These cells are unable adhere to each other, adhere to ECM substrates, unable to form clusters, survive, or grow in suspension due to the execution of efficient anoikis program^6^. Interestingly, our data suggest that B49Mod1 efficiently disrupts interaction between cells that express BST-2 dimers (BST-2D) but does not have effect on cells that express BST-2 monomers (BST-2M). Since B49Mod1 mediates similar effects as BST-2M cells^6^ and does not have an effect on BST-2M cells themselves (Fig. 8B,C,E), we propose that B49Mod1 functions by interfering with cancer cell interactions with other cells, as well as adhesion to target organ structures and substrates, such as matrix proteins. This hypothesis is supported by the finding that B49Mod1-treated cells are unable to efficiently adhere to fibronectin-treated plates (Fig. 3C).

Although B49/B49Mod1 meet pharmacological criteria for a contemporary drug lead, further studies are needed to fully characterize the peptide and identify proteases/peptidases that may deactivate the peptide. These studies should be followed by carefully designed plan to optimize the peptide for improved efficacy and determine the mechanism of action. Finally, we have shown that B49/B49Mod1 that mimics and binds the extracellular domain of BST-2 could be therapeutically useful in the treatment of breast cancer.

### Predictors of B49Mod1 response

About 67%, 25%, and 8.2% of breast tumors contain high, medium, or low levels of BST-2 protein^6^ while approximately 72% of all breast cancer subtypes express BST-2 mRNA^5,11^. Given that BST-2 is present in all breast tumors at different levels, and our results show that the effect of B49Mod1 is operative in all cancer cells tested, it seems that the role of BST-2 in breast cancer may be subtype independent and that B49Mod1 may be effective on all breast cancer subtypes. Therefore, a more robust study with multiple cell lines per cancer subtype is warranted to determine B49Mod1 specificity. The molecular diversity between the cell lines will allow this to be accomplished in a robust manner and establishing such associations is one of the first steps in B49Mod1-based drug development. In this study, we used a limited array of cell lines and observed that the biological response to B49Mod1 treatment does not depend on breast cancer subtype. Rather, we found that the presence of BST-2 in cells as well as the transformation (tumorigenic or non-tumorigenic) status of the cells are significant predictors of B49/B49Mod1 response. Thus, the convergence of a common cellular response for B49Mod1-targeting of similar oncogenic behavior in different cell lines of different subtypes substantiates the rationale for further development of B49Mod1 as an anti-adhesion and anti-cancer reagent, as well as potential treatment for breast cancer.

## Materials and Methods

### Animals

Five-week-old female BALB/cAnNCr mice (Harlan, Indianapolis, IN, USA) were used in all experiments. Experiments involving mice were approved by the University of Iowa Animal Care and Use Committee (IACUC). All experiments were performed in accordance with the approved University guidelines and regulations.

### Cell lines

4T1-luc cells were a kind gift from Dr. Lyse Norian of the University of Iowa. MDA-MB 231, T47D, AU565, BT-474, MDA-MB 134, MCF10a and HMLE cells were a kind gift of Dr. Weizhou Zhang from the University of Iowa. E0771 cells were purchased from CH3 BioSystems (Amherst, NY, USA). 4T1 and E0771 cells stably expressing a BST-2-targetin shRNA (shBST-2) or a non-targeting shRNA (shCTL) have been characterized previously^6,11^. Levels of BST-2 in 4T1 shBST-2 cells were rescued by stably expressing human wild-type (OE-BST-2D) or dimerization-deficient BST-2 (OE-BST-2D). Cells were maintained according to culture guidelines from the ATCC.

### Peptide synthesis

B49 is a 49-mer peptide which contains penetratin on its N-terminus was synthesized by Selleck Chemicals (Houston, TX, USA) with a purity of > 95%. The control peptide contains alanines in the place of cysteines and was also synthesized by Selleck Chemicals. B49Mod1 synthesized by ChinaPeptides (Shanghai, China) with a purity of > 95% is an analog of B49 were penetratin was removed and 4 amino acids were added, two on each terminus. Peptides were reconstituted in 1:8 acetonitrile to water to a final concentration of 1 mg/ml.

### Adhesion Assay

96-well plates were seeded with confluent monolayers of 4T1 or E0771 shCTL- or shBST-2-expressing cells. Incoming cells were labeled with PKH67Green fluorescent cell linker (Sigma-Aldrich) and 20,000 cells/well added to the monolayers and cells were allowed to incubate for 4 hours. Incoming cells that did not adhere were washed off with 1× PBS and plates were read using a Tecan Infinite M200 Pro plate reader (Tecan) at 485 nm/535 nm (excitation/emission) wavelengths to assess the degree of cell adhesion. Values for adhesion assays were plotted as relative fluorescence intensity (RFI). The variation of RFI values from experiment to experiment correspond to different uptake rates of PKH67 green dye by cells. For adhesion assays assessing the activity of B49 or B49Mod1, the aforementioned protocol was used except that cell monolayers were treated with 200 ng/well or another concentration as described in figures for 4 hours and then washed once with 1× PBS before the addition of PKH67Green-labeled cells. Adhesion assays in which Incoming cells were pre-treated with B49Mod1 were done in a similar way except that there was a 2 hour B49Mod1 pre-treatment step before PKH67Green-labeled cells were added to their respective cell monolayers, fibronectin-coated or B49-coated plates. 50 μl of fibronectin or B49Mod1 at a concentration of 50 μg/ml were used to pre-coat plates as previously described^11^.

### Intratumoral injections

Prior to intratumoral injections with B49, 300,000 4T1 cells expressing luciferase (Luc) were injected into the 10^th^ mammary fat pad and tumors were allowed to grow to a volume of 100 mm^3^. Tumor volume (TV) was calculated as previously described^6,11^. Intratumoral injections were performed every 3 days with 0.3 μg/μl of either B49 or control peptide at 0.33 μl/mm^3^ tumor. Tumor volume was measured in all animals prior to intratumoral injections and the concentration of B49 or the control peptide were adjusted appropriately. The anti-tumor activity of Control and B49 peptides was calculated by taking the average tumor volumes calculated between day 10 and day 35. We also calculated the percent of tumor growth inhibition by B49 over the Control peptide. To do this, we averaged tumor volumes from day 25 to 35 for each treatment group and then calculated tumor growth inhibition by dividing B49 average tumor volume by Control peptide average tumor volume and multiplying by 100.

### Viability Assay

Appropriate cell lines were seeded at 15,000 cells per well in a 96-well plate and allowed to adhere overnight. On the next day, cells were treated with B49Mod1 (500 ng/well) or a vehicle (Acetonitrile in water 1:8) for 24 hours. From this point on, a MTT assay was carried out as previously described^6,11^. Experiments were performed in replicates of at least six wells.

### Anchorage-independent Growth Assay

Colony formation assays were performed with several breast cancer cell lines using a previously described method^6,11^. B49Mod1 was used to treat cells at 500 ng/well and B49Mod1 was replenished every 3 days along with mew media. The experimental end-point was 30 days. Number of colonies was blind-counted manually under the microscope.

### RNA expression analyses

RNA isolation, cDNA synthesis and RT-qPCR were performed as previously described using published primers^5,6,11^. To analyze BST-2 transcript levels, all human samples were normalized to HMLE cells and murine cells were normalized to E0771 shControl (shCTL) cells.

### 3D multicellular spheroid assay in VitroGel 3D

10,000 PKH67Green-labeled 4T1 shBST-2 OE BST-2D or OE BST-2M cells were plated on 96-well plates in a 1:1 mixture of RPMI (with or without 500 ng/well of B49Mod1) with VitroGel 3D (TheWell Bioscience, Newark, NJ, USA) and were incubated for 10 days. The working solution of VitroGel 3D was previously diluted 2:1 in diH_2_O. Following addition of cells to a 96-well plate, the plate was incubated for 15 minutes at 37 °C to allow VitroGel 3D to solidify. To treat cells with B49Mod1, 500 ng/well of B49Mod1 were added in 100 μl of complete RPMI media on top of the layer of VitroGel 3D with cells. Media was replaced every 2 days until the experimental end point. Cells were imaged at 0, 1, 3, 7 and 10 days using a Nikon Eclipse Ti microscope adjusted with a Nikon digital sight camera (Nikon, Tokyo, Japan). Number of colonies was quantified manually.

### 3D monoculture spheroid assay

20,000 PKH67 green-labeled 4T1 shControl (shCTL) or shBST-2 cells were plated on 96-well plates previously coated with 12 mg/ml of Poly-HEMA at 50 µl/well as previously described [6]. Cells were treated with either Vehicle or 500 ng/well of B49Mod1 in 100 µl of complete RPMI. To form spheroids, the 96-well plate was centrifuged at 1200xg for 10 minutes at 37 °C. Spheroids were imaged at 0, 1, 2 and 3 days using a Nikon Eclipse Ti microscope adjusted with a Nikon digital sight camera (Nikon, Tokyo, Japan). Colony size and RFI of colonies were calculated using ImageJ.

### 3D coculture spheroid assay

10,000 PKH67Green-labeled 4T1 shControl (shCTL) or shBST-2 cells and 10,000 PKH26 red-labeled U937 cells were plated on 96-well plates previously coated with 12 mg/ml of Poly-HEMA at 50 µl/well as previously described^6^. Cell mixtures were treated with either Vehicle or 500 ng/well of B49Mod1 in 100 µl of complete RPMI. To form spheroids, 96-well plate was centrifuged at 1200xg for 10 minutes at 37 °C. Spheroids were imaged at 0, 1, 2 and 3 days using a Nikon Eclipse Ti microscope adjusted with a Nikon digital sight camera (Nikon, Tokyo, Japan).

### Statistics

Statistical analyses of significant differences were performed with unpaired t-test assuming Gaussian distribution with Welch’s correction (GraphPad Prism software, San Diego, CA, USA). Error bars represent standard deviation for RT-qPCR data and S.E.M. for other data. Kaplan–Meier survival plots were analyzed using the Gehan–Breslow–Wilcoxon test (GraphPad Prism software). A probability (P) value of 0.05 or lower was considered significant.
